# Performance of current ultrasound-based malignancy risk stratification systems for thyroid nodules in patients with follicular neoplasms

**DOI:** 10.1007/s00330-021-08450-3

**Published:** 2022-01-01

**Authors:** Yinghe Lin, Shuiqing Lai, Peiqing Wang, Jinlian Li, Zhijiang Chen, Long Wang, Haixia Guan, Jian Kuang

**Affiliations:** 1 Department of Endocrinology, Guangdong Provincial People’s Hospital, Guangdong Academy of Medical Sciences, Guangzhou, Guangdong China; 2 The Second School of Clinical Medicine, Southern Medical University, Guangzhou, Guangdong China; 3 Shantou University Medical College, Shantou, Guangdong China

**Keywords:** Thyroid neoplasms, Thyroid cancer, follicular, Ultrasonography, Biopsy, fine-needle

## Abstract

**Objectives:**

To investigate the ability of the currently used ultrasound-based malignancy risk stratification systems for thyroid neoplasms (ATA, AACE/ACE/AME, K-TIRADS, EU-TIRADS, ACR-TIRADS and C-TIRADS) in distinguishing follicular thyroid carcinoma (FTC) from follicular thyroid adenoma (FTA). Additionally, we evaluated the ability of these systems in correctly determining the indication for biopsy.

**Methods:**

Three hundred twenty-nine follicular neoplasms with definitive postoperative histopathology were included. The nodules were categorized according to each of six stratification systems, based on ultrasound findings. We dichotomized nodules into the positive predictive group of FTC (high and intermediate risk) and negative group of FTC based on the classification results. Missed biopsy was defined as neoplasms that were diagnosed as FTCs but for which biopsy was not indicated based on lesion classification. Unnecessary biopsy was defined as neoplasms that were diagnosed as FTAs but for whom biopsy was considered indicated based on classification. The diagnostic performance and missed and unnecessary biopsy rates were evaluated for each stratification system.

**Results:**

The area under the curve of each system for distinguishing follicular neoplasms was < 0.700 (range, 0.511–0.611). The missed biopsy rates were 9.0–22.4%. The missed biopsy rates for lesions ≤ 4 cm and lesions sized 2–4 cm were 16.2–35.1% and 0–20.0%, respectively. Unnecessary biopsy rates were 65.3–93.1%. In ≤ 4 cm group, the unnecessary biopsy rates were 62.2–89.7%.

**Conclusion:**

The malignancy risk stratification systems can select appropriate nodules for biopsy in follicular neoplasms, while they have limitations in distinguishing follicular neoplasms and reducing unnecessary biopsy. Specific stratification systems and recommendations should be established for follicular neoplasms.

**Key Points:**

*• Current ultrasound-based malignancy risk stratification systems of thyroid nodules had low efficiency in the characterization of follicular neoplasms.*

*• The adopted stratification systems showed acceptable performance for selecting FTC for biopsy but unsatisfactory performance for reducing unnecessary biopsy.*

**Supplementary Information:**

The online version contains supplementary material available at 10.1007/s00330-021-08450-3.

## Introduction

Both follicular thyroid adenoma (FTA) and follicular thyroid carcinoma (FTC) originate from follicular cells [1]. FTC is the second most common thyroid malignancy accounting for 10–15% of all malignant thyroid tumors. FTC shows a high propensity for hematogenous spread and 15% to 27% of these patients develop distant metastasis [2, 3]. Compared with papillary thyroid carcinoma (PTC), which is the most common subtype of thyroid cancer (approximately 80%), patients with FTC have a twofold higher risk of lung metastasis and tenfold higher risk of bone metastasis resulting in worse survival outcomes [2, 4, 5]. However, preoperative differentiation of FTC from its benign counterpart (FTA) is an inherently challenging aspect of management of thyroid nodules.

Ultrasound is the first-line imaging tool to evaluate the risk of malignancy and for formulating the optimal management strategy, including determining the indication for fine-needle aspiration (FNA), and informing treatment decision-making (surgical resection, monitoring, or no follow-up) [6]. Use of a single parameter for ultrasound evaluation may lead to inter-observer variability resulting in suboptimal sensitivity and specificity [7]. To standardize the evaluation of malignant thyroid nodules, various clinical societies have recently developed ultrasound-based malignancy risk stratification systems [8–13]. Based on the different versions of the Thyroid Imaging Reporting and Data System (TIRADS), several “pattern-based” systems and “score-based” systems have been established. The former are represented by 2015 ATA (American Thyroid Association), AACE/ACE/AME (American Association of Clinical Endocrinologists, American College of Endocrinology, and Associazione Medici Endocrinology), K-TIRADS (Korean Society of Thyroid Radiology), and EU-TIRADS (European Thyroid Association). The latter include ACR-TIRADS (American College of Radiology) and C-TIRADS (2020 Chinese Guidelines for Ultrasound Malignancy Risk Stratification of Thyroid Nodules). All these systems have shown reliable performance for the diagnosis and for selecting candidates for FNA [14–17].

As mentioned above, there are considerable differences in the incidence rates of various thyroid cancer subtypes [2]. In previous studies that evaluated these systems, the vast majority of malignant specimens were PTCs (88.9 to 99.6%) [18], which may have introduced an element of bias. Ultrasonographic features of FTC and PTC are considerably different [3]. The established malignant features, including hypoechogenicity, irregular margins, microcalcifications, and nonparallel orientation, are more common in PTC than in FTC.

Currently, few studies have focused on the value of ultrasound-based malignancy risk stratification systems in patients with follicular neoplasms [19–21]. The performance of these systems in distinguishing FTC from FTA and in correctly determining the indications for biopsy is uncertain. Thus, we hypothesized that the relevant conclusions reported in the past could not be simply extrapolated to follicular tumors.

In this study, we aimed to investigate the performance of the currently used systems in the context of follicular neoplasms. For this purpose, we compared the ability of the current systems in distinguishing FTC from FTA. Additionally, we assessed whether these systems can help identify the nodules that require a biopsy and can reduce the rate of unnecessary biopsies.

## Materials and methods

This retrospective study was approved by the Institutional Review Board, and the requirement for informed consent to review images and medical records was waived.

### Patients

From January 2014 to May 2020, 441 consecutive patients (455 nodules) with thyroid follicular neoplasms proven by histopathological examination of the surgical specimens following thyroidectomy at a tertiary referral center were included in this study. Fourteen patients were pathologically confirmed to have two lesions each (4 with FTCs and 10 with FTAs). For these patients, the larger nodule among the two lesions was selected. The exclusion criteria were as follows: absence of preoperative thyroid US images (*n* = 55); uncertain match between imaging findings and histopathological results (*n* = 8). Because hyperfunctioning nodules do not require FNA [8], definitive or suspected hot nodules were also excluded (*n* = 49).

### Ultrasonography

Ultrasonography examinations of thyroid glands and cervical regions were performed with Aplio 500 (Toshiba Medical System), HI Vision Ascendus (Hitachi Medical Corporation), or HI Vision Preirus (Hitachi Medical Corporation) ultrasound instruments equipped with 5–12-MHz linear array transducers. The following ultrasound features of each neoplasm were recorded and reviewed by 2 researchers who were blinded to the diagnosis: maximum diameter (cm); location (left, right, or isthmus); composition (spongiform, cystic, mixed, or solid); echogenicity (anechoic, hyperechoic, isoechoic, hypoechoic, or very hypoechoic); margin (smooth, ill-defined, or irregular); calcification (absent, microcalcification, macrocalcification, or rim calcification); shape (round to oval or irregular); orientation in transverse view (parallel or nonparallel); hypoechoic peripheral halo (absence or presence); vascularization (absent, perinodular, intranodular, or mixed); and the location of the solid component for mixed-content nodules (eccentric or non-eccentric). The presence of other hyperechoic foci (comet-tail artifacts or indeterminate), extrathyroidal extension, and suspicious cervical lymph node was also investigated. Any disagreement between the 2 researchers with respect to these features was resolved by consensus.

### Categorization according to the risk stratification systems

Based on retrospective analysis of ultrasound features, each thyroid nodule was categorized using the six stratification systems: 2015 ATA, AACE/ACE/AME, K-TIRADS, EU-TIRADS, ACR-TIRADS, and C-TIRADS [8–13]. For statistical analysis, firstly, the nodules were dichotomized into the positive predictive group of FTC (high and intermediate suspicion according to 2015 ATA; high and intermediate risk according to AACE/ACE/AME; high and intermediate suspicion according to K-TIRADS; high and intermediate risk according to EU-TIRADS; category 4 and 5 according to ACR TI-RADS; category 4B to 5 according to C-TIRADS) and negative predictive group of FTC (benign, very low, and low suspicion according to 2015 ATA; low risk according to AACE/ACE/AME; benign and low suspicion according to K-TIRADS; benign and low risk according to EU-TIRADS; category 1 to 3 according to ACR-TIRADS; category 2 to 4A according to C-TIRADS).

Secondly, based on the risk stratification recommendations, the nodules were retrospectively divided into 2 categories: “indication for FNA” and “no indication for FNA” (Supplementary 1). Missed biopsy was defined as any case of FTC nodule for which FNA was not indicated based on the risk stratification system. Unnecessary biopsy was defined as any case of FTA for which FNA was considered indicated based on the risk stratification system. Missed biopsy rate and unnecessary biopsy rate were calculated for each of the six systems.

In addition, nodules that did not conform to any category according to the systems were included in the non-classifiable group. For non-classifiable nodules, FNA was only considered to be recommended if suspicious cervical lymph nodes were present.

### Data and statistical analysis

Nominal and ordinal variables are expressed as frequencies and proportions, while continuous variables were expressed as mean ± standard deviation (SD) and range. Between-group differences with respect to demographic, clinical, and ultrasound features were assessed using independent two-sample *t* test or rank-sum test for continuous variables and chi-square test or Fisher’s exact test for nominal variables. Data pertaining to the distribution of lesions in various categories according to the risk stratification systems were analyzed using Mann–Whitney *U* test or Kruskal–Wallis test for ordinal variables. Based on the established cutoff, diagnostic performance of the systems was assessed using receiver operating characteristic (ROC) curve analysis. Sensitivity, specificity, positive predictive value (PPV), negative predictive value (NPV), and area under the ROC curves (AUC) were calculated with 95% confidence intervals (95% CI). AUCs of each system were compared using the DeLong method. Missed biopsy rates and unnecessary biopsy rates were compared using Cochran’s Q test, respectively. All statistical analyses were performed using SPSS (version 23.0, IBM) or MedCalc software (version 19.0.4) software. Two-sided *p* values < 0.05 were considered indicative of statistical significance.

## Results

A total of 329 patients (329 nodules) were included in this study. These included 216 (65.7%) women and 113 (34.3%) men; the mean age of patients was 43.5 ± 14.3 years (range, 3–82).

### Baseline

Of the 329 follicular neoplasms, 262 (79.6%) were diagnosed as FTAs and 67 (20.4%) were diagnosed as FTCs based on histopathological examination of surgical specimens The mean maximum diameter of the follicular neoplasms was 3.6 ± 1.7 cm (range, 0.6–12.1). The most common ultrasound presentation of follicular neoplasm in our cohort was solid (76.3%), isoechoic (79.0%), smooth margin (92.1%), non-calcification (85.7%), round to oval (96.0%), and parallel (99.7%) nodule with peripheral halo (59.0%) and mixed vascularization (93.1%) (Table 1). There were no significant differences between FTA and FTC with respect to age, sex, maximum diameter, nodule location, orientation, peripheral halo, extrathyroidal extension, or other hyperechoic foci (comet-tail artifacts or indeterminate) (*p* > 0.05). However, there were significant differences with respect to following ultrasound features: hypoechoic (15.3% vs 26.9%, *p* = 0.026), ill-defined margin (4.2% vs 20.9%, *p* < 0.001), calcifications (10.7% vs 20.84%, *p* < 0.001), microcalcifications (3.1% vs 11.9%, *p* = 0.003), irregular shape (1.1% vs 14.9%, *p* < 0.001), suspicious cervical lymph node (0% vs 4.5%, *p* = 0.008), and the location of the solid component in mixed-content nodules (25.4% vs 80.0%, *p* = 0.023) (detailed comparisons among FTC subtypes in Supplementary 2).

**Table 1:** Demographic characteristics and ultrasound features of 329 follicular thyroid neoplasms

Demographic characteristics		No. (%) or mean ± SD (range)
Male		113 (34.3)
Age (years)		43.5 ± 14.3 (3–82)
Pathological classifications		
FTA		262 (79.6)
FTC		67 (20.4)
Minimally invasive		48 (14.7)
Encapsulated angioinvasive		12 (3.6)
Widely invasive		7 (2.1)
Ultrasound features		
Maximum diameters (cm)^a^		3.6 ± 1.7 (0.6–12.1)
Maximum diameters > 2 cm^a^		285 (86.9)
Maximum diameters > 4 cm^a^		135 (41.2)
Location	Left	167 (50.8)
	Right	157 (47.7)
	Isthmus	5 (1.5)
Composition	Spongiform	4 (1.2)
	Cystic	0 (0)
	Mixed	72 (21.9)
	Solid	251 (76.3)
	Undetermined because of calcification	2 (0.6)
Echogenicity^c^	Anechoic	0 (0)
	Hyperechoic	11 (3.4)
	Isoechoic	260 (79.0)
	Hypoechoic	58 (17.6)
	Very hypoechoic	0 (0)
Margin	Smooth	303 (92.1)
	Ill-defined	25 (7.6)
	Irregular	1 (0.3)
Calcifications^b^	Absent	282 (85.7)
	Microcalcification	16 (4.9)
	Macrocalcification	26 (7.9)
	Rim calcification	7 (2.1)
Shape	Round to oval	316 (96.0)
	Irregular	13 (4.0)
Orientation	Parallel	328 (99.7)
	Nonparallel	1 (0.3)
Peripheral halo	Present	194 (59.0)
Hyperechoic foci	Comet-tail artifacts	4 (1.2)
	Indeterminate	3 (0.9)
Extrathyroidal extension	Present	1 (0.3)
Suspicious cervical lymph node	Present	3 (0.9)
The location of the solid component for mixed-content nodules	Eccentric	21 (29.2)
	Non-eccentric	51 (70.8)
Vascularization	Absent	11 (3.3)
	Perinodular	6 (1.8)
	Intranodular	6 (1.8)
	Mixed	306 (93.1)
Ultrasound-based malignancy risk stratification systems		
ATA	Benign	0 (0)
	Very low suspicion	53 (16.1)
	Low suspicion	213 (64.7)
	Intermediate suspicion	37 (11.3)
	High suspicion	8 (2.4)
	Nonclassifiable group	18 (5.5)
AACE/ACE/AME	Low	11 (3.4)
	Intermediate	293 (89.0)
	High suspicion	18 (5.5)
	Nonclassifiable group	7 (2.1)
K-TIRADS	Benign (K-TR2)	8 (2.4)
	Low suspicion (K-TR3)	258 (78.5)
	Intermediate suspicion (K-TR4)	53 (16.1)
	High suspicion (K-TR5)	8 (2.4)
	Nonclassifiable group	12 (3.6)
EU-TIRADS	Benign (EU-TR2)	4 (1.2)
	Low risk (EU-TR3)	252 (76.5)
	Intermediate risk (EU-TR4)	38 (11.6)
	High risk (EU-TR5)	24 (7.3)
	Nonclassifiable group	11 (3.4)
ACR-TIRADS	Benign (ACR-TR1)	4 (1.2)
	Not suspicious (ACR-TR2)	62 (18.8)
	Mildly suspicious (ACR-TR3)	185 (56.3)
	Moderately suspicious (ACR-TR4)	70 (21.3)
	Highly Suspicious (ACR-TR5)	8 (2.4)
C-TIRADS	C-TR2	8 (2.4)
	C-TR3	67 (20.4)
	C-TR4A	224 (68.1)
	C-TR4B	19 (5.8)
	C-TR4C	8 (2.4)
	C-TR5	3 (0.9)

^a^The maximum diameters could not be determined in a case due to macrocalcifications

^b^Some nodules presented multiple types of calcifications

^c^Hyperechoic, isoechoic and hypoechoic: compared to adjacent parenchyma; very hypoechoic: more hypoechoic than strap muscles

*Abbreviations*: *FTA* follicular thyroid adenoma; *FTC* follicular thyroid carcinoma; *ATA* 2015 American Thyroid Association Management Guidelines for Adult Patients with Thyroid Nodules and Differentiated Thyroid Cancer; *AACE/ACE/AME* American Association of Clinical Endocrinologists, American College of Endocrinology, and Associazione Medici Endocrinology Medical Guidelines for Clinical Practice for the Diagnosis and Management of Thyroid Nodules (2016 Update); *EU-TIRADS* European Thyroid Association Guidelines for Ultrasound Malignancy Risk Stratification of Thyroid Nodules in Adults; *K-TIRADS* Revised Korean Society of Thyroid Radiology Consensus Statement and Recommendations; *ACR-TIRADS* American College of Radiology Thyroid Imaging Reporting and Data System; *C-TIRADS* 2020 Chinese Guidelines for Ultrasound Malignancy Risk Stratification of Thyroid Nodules

The most frequent classification was low suspicion by 2015 ATA (64.7%), intermediate risk by AACE/ACE/AME (89.0%), low suspicion by K-TIRADS (78.5%), low risk by EU-TIRADS (76.5%), TR3 by ACR-TIRADS (56.3%), and TR4A by C-TIRADS (68.1%) (Table 1). Statistical comparisons between FTA and FTC showed significant differences in distributions according to the six systems respectively, while no significant difference was found among FTC subtypes (Table 2).

**Table 2:** Distribution of follicular thyroid neoplasms among the ultrasound-based malignancy risk stratification systems

		FTA	FTC	*p*	Minimally invasive	Encapsulated angioinvasive	Widely invasive	*p*
Ultrasound-based malignancy risk stratification systems								
ATA	Total classification	251	60	*p* < 0.001	42	12	6	*p* = 0.870
	Benign	0 (0)	0 (0)		0 (0)	0 (0)	0 (0)	
	Very low suspicion	52 (20.7)	1 (1.7)		1 (2.4)	0 (0)	0 (0)	
	Low suspicion	168 (66.9)	45 (75.0)		31 (73.8)	9 (75.0)	5 (83.3)	
	Intermediate suspicion	28 (11.2)	9 (15.0)		7 (16.7)	1 (8.3)	1 (16.7)	
	High suspicion	3 (1.2)	5 (8.3)		3 (7.1)	2 (16.7)	0 (0)	
AACE/ACE/AME	Total classification	260	62	*p* < 0.001	44	12	6	*p* = 0.577
	Low-risk	10 (3.8)	1 (1.6)		1 (2.3)	0 (0)	0 (0)	
	Intermediate-risk	242 (93.1)	51 (82.3)		37 (84.1)	9 (75.0)	5 (83.3)	
	High-risk	8 (3.1)	10 (16.1)		6 (13.6)	3 (25.0)	1 (16.7)	
K-TIRADS	Total classification	261	66	*p* = 0.002	48	12	6	*p* = 0.685
	Benign	8 (3.1)	0 (0)		0 (0)	0 (0)	0 (0)	
	Low suspicion	212 (81.2)	46 (69.7)		32 (66.7)	9 (75.0)	5 (83.3)	
	Intermediate suspicion	38 (14.6)	15 (22.7)		13 (27.0)	1 (8.3)	1 (16.7)	
	High suspicion	3 (1.1)	5 (7.6)		3 (6.3)	2 (16.7)	0 (0)	
EU-TIRADS	Total classification	256	62	*p* < 0.001	46	10	6	*p* = 0.686
	Benign	4 (1.6)	0 (0)		0 (0)	0 (0)	0 (0)	
	Low risk	211 (82.4)	41 (66.1)		31 (67.4)	7 (70.0)	3 (50.0)	
	Intermediate risk	31 (12.1)	7 (11.3)		5 (10.9)	1 (10.0)	1 (16.7)	
	High risk	10 (3.9)	14 (22.6)		10 (21.7)	2 (20.0)	2 (33.3)	
ACR-TIRADS	Total classification	262	67	*p* < 0.001	48	12	7	*p* = 0.736
	ACR-TR1	4 (1.5)	0 (0)		0 (0)	0 (0)	0 (0)	
	ACR-TR2	58 (22.1)	4 (6.0)		4 (8.3)	0 (0)	0 (0)	
	ACR-TR3	148 (56.6)	37 (55.2)		25 (52.1)	7 (58.3)	5 (71.4)	
	ACR-TR4	49 (18.7)	21 (31.3)		16 (33.3)	3 (25.0)	2 (28.6)	
	ACR-TR5	3 (1.1)	5 (7.5)		3 (6.3)	2 (16.7)	0 (0)	
C-TIRADS	Total classification	262	67	*p* < 0.001	48	12	7	*p* = 0.075
	C-TR2	8 (3.1)	0 (0)		0 (0)	0 (0)	0 (0)	
	C-TR3	61 (23.3)	6 (9.0)		6 (12.5)	0 (0)	0 (0)	
	C-TR4A	181 (69.1)	43 (64.2)		32 (66.7)	7 (58.3)	4 (57.1)	
	C-TR4B	9 (3.4)	10 (14.9)		6 (12.5)	2 (16.7)	2 (28.6)	
	C-TR4C	3 (1.1)	5 (7.5)		4 (8.3)	1 (8.3)	0 (0)	
	C-TR5	0 (0)	3 (4.5)		0 (0)	2 (16.7)	1 (14.3)	

Values are presented as number (%)

*Abbreviations*: *FTA* follicular thyroid adenoma; *FTC* follicular thyroid carcinoma; *ATA* 2015 American Thyroid Association Management Guidelines for Adult Patients with Thyroid Nodules and Differentiated Thyroid Cancer; *AACE/ACE/AME* American Association of Clinical Endocrinologists, American College of Endocrinology, and Associazione Medici Endocrinology Medical Guidelines for Clinical Practice for the Diagnosis and Management of Thyroid Nodules (2016 Update); *EU-TIRADS* European Thyroid Association Guidelines for Ultrasound Malignancy Risk Stratification of Thyroid Nodules in Adults; *K-TIRADS* Revised Korean Society of Thyroid Radiology Consensus Statement and Recommendations; *ACR-TIRADS* American College of Radiology Thyroid Imaging Reporting and Data System; *C-TIRADS* 2020 Chinese Guidelines for Ultrasound Malignancy Risk Stratification of Thyroid Nodules

### Performance of the stratification systems

The diagnostic indices of the six systems depending on predictive classifications are presented in Table 3. The AUCs for the six systems ranged from 0.511 to 0.611. K-TIRADS, EU-TIRADS, ACR-TIRADS, and C-TIRADS indicated statistical value for FTC (AUCs = 0.573–0.611, *p* < 0.05). The AUC of C-TIRADS for differentiating FTC from FTA was the highest among all systems (0.611; 95% CI, 0.556–0.664) (sensitivity: 26.9% (95% CI, 16.8 to 39.1%); specificity: 95.4% (95% CI, 92.1 to 97.6%); PPV: 60.0% (95% CI, 43.2 to 74.7%); NPV: 83.6% (95% CI, 81.5 to 85.5%)). AUCs of the statistical value systems were no significant difference (*p* > 0.05).

**Table 3:** Diagnostic indices of the systems for follicular thyroid neoplasms depending on predictive classifications. Classifiable nodules of each system were included

Ultrasound-based malignancy risk stratification systems	Sensitivity(%)	Specificity(%)	PPV(%)	NPV(%)	AUC	*p*
ATA	23.3 (13.4–36.0)	87.7 (82.9–91.5)	31.1 (20.4–44.3)	82.7 (80.5–84.7)	0.555 (0.498–0.611)	*p* = 0.062
AACE/ACE/AME	98.4 (91.3–99.9)	3.84 (1.9–7.0)	19.6 (19.0–20.3)	90.9 (56.6–98.7)	0.511 (0.455–0.567)	*p* = 0.266
K-TIRADS	30.3 (19.6–42.9)	84.3 (79.3–88.5)	32.7 (23.5–43.6)	82.7 (80.2–85.0)	0.573 (0.517–0.627)	*p* = 0.017^a^
EU-TIRADS	33.9 (22.3–47.0)	84.0 (78.9–88.3)	33.9 (24.7–44.5)	84.0 (81.3–86.3)	0.589 (0.533–0.644)	*p* = 0.006^a^
ACR-TIRADS	38.8 (27.1–51.5)	80.2 (74.8–84.8)	33.3 (25.4–42.4)	83.7 (80.7–86.2)	0.595 (0.540–0.648)	*p* = 0.004^a^
C–TIRADS	26.9 (16.8–39.1)	95.4 (92.1–97.6)	60.0 (43.2–74.7)	83.6 (81.5–85.5)	0.611 (0.556–0.664)	*p* < 0.001^a^

Numbers in parentheses are 95% confidence intervals

^a^There was no significant difference between the AUCs of the above four systems (*p* > 0.05)

*Abbreviations*: *PPV* positive predictive value; *NPV* negative predictive value; *ATA* 2015 American Thyroid Association Management Guidelines for Adult Patients with Thyroid Nodules and Differentiated Thyroid Cancer; *AACE/ACE/AME* American Association of Clinical Endocrinologists, American College of Endocrinology, and Associazione Medici Endocrinology Medical Guidelines for Clinical Practice for the Diagnosis and Management of Thyroid Nodules (2016 Update); *EU-TIRADS* European Thyroid Association Guidelines for Ultrasound Malignancy Risk Stratification of Thyroid Nodules in Adults; *K-TIRADS* Revised Korean Society of Thyroid Radiology Consensus Statement and Recommendations; *ACR-TIRADS* American College of Radiology Thyroid Imaging Reporting and Data System; *C-TIRADS* 2020 Chinese Guidelines for Ultrasound Malignancy Risk Stratification of Thyroid Nodules

Notably, of the 27 non-classifiable nodules (8.2%, 27 of 329) according to 1 or more systems, 14 (20.9%, 14 of 67) were FTCs and 13 (5.0%, 13 of 262) were FTAs (Table 4). All cases were classifiable according to ACR-TIRADS and C-TIRADS.

**Table 4:** Unclassifiable follicular thyroid neoplasms according to the ultrasound-based malignancy risk stratification systems

Nodule ID	Pathological diagnosis	Ultrasound description	Maximum diameters (cm)	Unclassified by					
				ATA	AACE/ACE/AME	K-TIRADS	EU-TIRADS	ACR-TIRADS	C-TIRADS
4	FTC (minimally invasive)	Solid, hypoechoic, ill-defined margin, irregular shape, macrocalcifications	1.7	X	X				
23	FTC (minimally invasive)	Solid, hypoechoic, ill-defined margin, round to oval shape, peripheral halo	2.7	X			X		
33	FTC (minimally invasive)	Solid, isoechoic, smooth margin, round to oval shape, microcalcififications, peripheral halo	2.7	X					
35	FTC (minimally invasive)	Solid, isoechoic, ill-defined margin, irregular shape, peripheral calcifications, peripheral halo	4.1		X				
36	FTC (widely invasive)	Solid, hypoechoic, ill-defined margin, irregular shape	4.7		X				
38	FTC (minimally invasive)	Solid, isoechoic, ill-defined margin, round to oval shape, microcalcififications, peripheral halo	2.9	X					
39	FTC (widely invasive)	Composition cannot be determined because of macrocalcifications, present a suspicious cervical lymph node	Undetermined because of macrocalcifications	X		X			
41	FTC (minimally invasive)	Solid, hypoechoic, smooth margin, irregular shape, peripheral halo	2.0		X				
42	FTC (minimally invasive)	Solid, hyperechoic, smooth margin, round to oval shape, microcalcififications, peripheral halo	3.0	X					
44	FTC (widely invasive)	Solid, isoechoic, ill-defined margin, round to oval shape	2.4				X		
46	FTC (minimally invasive)	Solid, hypoechoic, ill-defined margin, round to oval shape, macrocalcifications, peripheral halo	2.7	X			X		
49	FTC (encapsulated angioinvasive)	Solid, isoechoic, ill-defined margin, round to oval shape, peripheral halo	12.1				X		
61	FTC (encapsulated angioinvasive)	Solid, isoechoic, ill-defined margin, round to oval shape, macrocalcifications	5.7				X		
94	FTC (minimally invasive)	Solid, hypoechoic, smooth margin, irregular shape	5.7		X				
73	FTA	Solid, isoechoic, ill-defined margin, round to oval shape, macrocalcifications	9.0				X		
74	FTA	Solid, isoechoic, ill-defined margin, irregular shape, microcalcififications	2.0	X					
81	FTA	Composition cannot be determined because of peripheral calcifications	4.4	X	X	X			
210	FTA	Solid, hypoechoic, ill-defined margin, round to oval shape	0.8	X			X		
223	FTA	Mixed, isoechoic, ill-defined margin, round to oval shape, macrocalcifications, peripheral halo	2.8				X		
241	FTA	Solid, isoechoic, smooth margin, round to oval shape, microcalcifications, peripheral halo	2.5	X					
254	FTA	Solid, hypoechoic, ill-defined margin, round to oval shape	1.1	X			X		
275	FTA	Mixed, isoechoic, smooth margin, round to oval shape, microcalcifications	2.9	X					
284	FTA	Solid, hypoechoic, ill-defined margin, irregular shape	3.5	X	X				
293	FTA	Solid, hypoechoic, ill-defined margin, round to oval shape, peripheral calcifications	1.1	X			X		
295	FTA	Solid, isoechoic, ill-defined margin, round to oval shape, microcalcifications	0.7	X					
313	FTA	Solid, hypoechoic, ill-defined margin, round to oval shape	1.1	X			X		
324	FTA	Mixed, isoechoic, smooth margin, round to oval shape, microcalcifications	4.1	X					

A nodule was undetermined composition without any high-risk features, which could not be classified according to 2015 ATA, AACE/ACE/AME and K-TIRADS

A nodule was undetermined composition with a suspicious cervical lymph node, which could not be classified according to 2015 ATA and K-TIRADS

Six nodules were irregular shape without any high-risk features, which could not be classified according to AACE/ACE/AME

Eight nodules were iso/hyperechoic with microcalcification, which could not be classified according to 2015 ATA

Eight solid nodules were hypoechoic with ill-defined margin, which could not be classified according to 2015 ATA

Eleven nodules were ill-defined margin without any high-risk features, which could not be classified according to EU-TIRADS

*Abbreviations*: *FTA* follicular thyroid adenoma; *FTC* follicular thyroid carcinoma; *ATA* 2015 American Thyroid Association Management Guidelines for Adult Patients with Thyroid Nodules and Differentiated Thyroid Cancer; *AACE/ACE/AME* American Association of Clinical Endocrinologists, American College of Endocrinology, and Associazione Medici Endocrinology Medical Guidelines for Clinical Practice for the Diagnosis and Management of Thyroid Nodules (2016 Update); *EU-TIRADS* European Thyroid Association Guidelines for Ultrasound Malignancy Risk Stratification of Thyroid Nodules in Adults; *K-TIRADS* Revised Korean Society of Thyroid Radiology Consensus Statement and Recommendations; *ACR-TIRADS* American College of Radiology Thyroid Imaging Reporting and Data System; *C-TIRADS* 2020 Chinese Guidelines for Ultrasound Malignancy Risk Stratification of Thyroid Nodules

The performance of the six systems in correctly determining the indication for biopsy and reducing unnecessary biopsy is shown in Table 5. The lowest missed biopsy rate was found with K-TIRADS (9.0%), and the highest with ACR-TIRADS and AACE/ACE/AME (22.4%). The missed biopsy rates were significantly different between the six systems (*p* = 0.049), but not in pairwise comparisons. ACR-TIRADS was associated with the lowest unnecessary biopsy rate (65.3%), while K-TIRADS was associated with the highest unnecessary biopsy rate (93.1%). The unnecessary biopsy rates were significantly different among the six systems (*p* < 0.001). The unnecessary biopsy rates of ACR-TIRADS (65.3%) and C-TIRADS (67.9%) were lower than those of the other systems.

**Table 5:** Ability of the ultrasound-based malignancy risk stratification systems to select proper nodules for biopsy and reduce unnecessary biopsy

Ultrasound-based malignancy risk stratification systems		Missed biopsy^b^	Missed biopsy (≤ 4 cm)^c^	Missed biopsy (2–4 cm)^d^		Unnecessary biopsy^e^	Unnecessary biopsy (≤ 4 cm)^f^
ATA	Benign	0/0 (0)	0/0 (0)	0/0 (0)		0/0 (0)	0/0 (0)
	Very low suspicion	0/1 (0)	0/1 (0)	0/1 (0)		49/52 (92.3)	24/27 (88.9)
	Low suspicion	3/45 (6.7)	3/23 (13.0)	0/17 (0)		161/168 (95.8)	90/97 (92.8)
	Intermediate suspicion	1/9 (11.1)	1/4 (25.0)	0/1 (0)		24/28 (85.7)	18/22 (81.8)
	High suspicion	2/5 (40.0)	2/3 (66.7)	0/1 (0)		3/3 (100)	1/1 (100)
	Not classifiable	6/7 (85.7)^a^	6/6 (100)	5/5 (100)		0/11 (0)	0/9 (0)
	Total	12/67 (17.9)	12/37 (32.4)	5/25 (20.0)		237/262 (90.5)	133/156 (85.3)
AACE/ACE/AME	Low	0/1 (0)	0/0 (0)	0/0 (0)		8/10 (80.0)	6/8 (75.0)
	Intermediate	8/51 (15.7)	8/28 (28.6)	0/20 (0)		216/242 (89.3)	116/142 (81.7)
	High suspicion	2/10 (20.0)	2/7 (28.6)	0/5 (0)		7/8 (87.5)	4/5 (80.0)
	Not classifiable	5/5 (100)	2/2 (100)	0/0 (0)		0/2 (0)	0/1 (0)
	Total	15/67 (22.4)	12/37 (32.4)	0/25 (0)		231/262 (88.2)	126/156 (80.8)
K-TIRADS	Benign	0/0 (0)	0/0 (0)	0/0 (0)		4/8 (50.0)	4/7 (57.1)
	Low suspicion	3/46 (6.5)	3/23 (13.0)	0/18 (0)		205/212 (96.7)	107/114 (93.9)
	Intermediate suspicion	1/15 (6.7)	1/11 (9.1)	0/6 (0)		32/38 (84.2)	28/34 (82.4)
	High suspicion	2/5 (40.0)	2/3 (66.7)	0/1 (0)		3/3 (100)	1/1 (100)
	Not classifiable	0/1 (0)^a^	0/0 (0)	0/0 (0)		0/1 (0)	0/0 (0)
	Total	6/67 (9.0)	6/37 (16.2)	0/25 (0)		244/262 (93.1)	140/156 (89.7)
EU-TIRADS	Benign	0/0 (0)	0/0 (0)	0/0 (0)		0/4 (0)	0/4 (0)
	Low risk	6/41 (14.6)	6/22 (27.3)	0/17 (0)		195/211 (92.4)	100/116 (86.2)
	Intermediate risk	1/7 (14.3)	1/4 (25.0)	0/1 (0)		26/31 (83.9)	20/25 (80.0)
	High risk	2/14 (14.3)	2/8 (25.0)	0/4 (0)		9/10 (90.0)	5/6 (83.3)
	Not classifiable	5/5 (100)	3/3 (100)	3/3 (100)		0/6 (0)	0/5 (0)
	Total	14/67 (20.9)	12/37 (32.4)	3/25 (12.0)		230/262 (87.8)	125/156 (80.1)
ACR-TIRADS	ACR-TR1	0/0 (0)	0/0 (0)	0/0 (0)		0/4 (0)	0/4 (0)
	ACR-TR2	4/4 (100)	2/2 (100)	2/2 (100)		0/58 (0)	0/26 (0)
	ACR-TR3	8/37 (21.6)	8/19 (42.1)	2/13 (15.4)		129/148 (87.2)	70/89 (78.7)
	ACR-TR4	1/21 (4.8)	1/13 (7.7)	0/9 (0)		39/49 (79.6)	26/36 (72.2)
	ACR-TR5	2/5 (40.0)	2/3 (66.7)	0/1 (0)		3/3 (100)	1/1 (100)
	Total	15/67 (22.4)	13/37 (35.1)	4/25 (16.0)		171/262 (65.3)	97/156 (62.2)
C-TIRADS	C-TR2	0/0 (0)	0/0 (0)	0/0 (0)		0/8 (0)	0/7 (0)
	C-TR3	6/6 (100)	4/4 (100)	4/4 (100)		0/61 (0)	0/24 (0)
	C-TR4A	5/43 (11.6)	5/22 (22.7)	0/13 (0)		168/181 (92.8)	103/116 (88.8)
	C-TR4B	0/10 (0)	0/6 (0)	0/5 (0)		8/9 (88.9)	5/6 (83.3)
	C-TR4C	2/5 (60.0)	2/4 (50.0)	0/2 (0)		2/3 (66.7)	2/3 (66.7)
	C-TR5	0/3 (0)	0/1 (0)	0/1 (0)		0/0 (0)	0/0 (0)
	Total	13/67 (19.4)	11/37 (29.7)	4/25 (16.0)		178/262 (67.9)	110/156 (70.5)

Values are presented as number (%)

All *p* values have been adjusted in pairwise comparisons

^a^No.39 (Table 4): an unclassifiable nodule with unknown size was indicated for FNA due to a suspicious cervical lymph node according to ATA and K-TIRADS

^b^The missed biopsy rates were significantly different among the six systems (*p* = 0.049), but not found in pairwise comparisons

^c^There was no significant difference between the six systems with respect to missed biopsy rate (≤ 4 cm) (*p* = 0.135)

^d^There was no significant difference between the six systems with respect to missed biopsy rate (2–4 cm) (*p* = 0.075)

^e^The unnecessary biopsy rates were significantly different among the six systems (*p* < 0.001): ACR-TIRADS vs. AACE/ACE/AME, *p* < 0.001; ACR-TIRADS vs. EU-TIRADS, *p* < 0.001; ACR-TIRADS vs. K-TIRADS, *p* < 0.001; ACR-TIRADS vs. ATA, *p* < 0.001; C-TIRADS vs. AACE/ACE/AME, *p* < 0.001; C-TIRADS vs. EU-TIRADS, *p* < 0.001; C-TIRADS vs. K-TIRADS, *p* < 0.001; C-TIRADS vs. ATA, *p* < 0.001

^f^The unnecessary biopsy rates (≤ 4 cm) were significantly different among the six systems (*p* < 0.001): ACR-TIRADS vs. AACE/ACE/AME, *p* < 0.001; ACR-TIRADS vs. EU-TIRADS, *p* < 0.001; ACR-TIRADS vs. K-TIRADS, *p* < 0.001; ACR-TIRADS vs. ATA, *p* < 0.001; C-TIRADS vs. AACE/ACE/AME, *p* = 0.024; C-TIRADS vs. EU-TIRADS, *p* = 0.046; C-TIRADS vs. K-TIRADS, *p* < 0.001; C-TIRADS vs. ATA, *p* < 0.001; EU-TIRADS vs. K-TIRADS, *p* = 0.046

*Abbreviations*: *FNA* fine-needle aspiration; *FTA* follicular thyroid adenoma; *FTC* follicular thyroid carcinoma; *ATA* 2015 American Thyroid Association Management Guidelines for Adult Patients with Thyroid Nodules and Differentiated Thyroid Cancer; *AACE/ACE/AME* American Association of Clinical Endocrinologists, American College of Endocrinology, and Associazione Medici Endocrinology Medical Guidelines for Clinical Practice for the Diagnosis and Management of Thyroid Nodules (2016 Update); *EU-TIRADS* European Thyroid Association Guidelines for Ultrasound Malignancy Risk Stratification of Thyroid Nodules in Adults; *K-TIRADS* Revised Korean Society of Thyroid Radiology Consensus Statement and Recommendations; *ACR-TIRADS* American College of Radiology Thyroid Imaging Reporting and Data System; *C-TIRADS* 2020 Chinese Guidelines for Ultrasound Malignancy Risk Stratification of Thyroid Nodules

We further performed sub-group analysis based on nodule size. The missed biopsy rates for lesions ≤ 4 cm ranged from 16.2 to 35.1%; the missed biopsy rates for lesions sized 2–4 cm ranged from 0 to 20.0%; there was no significant difference between the six systems with respect to missed biopsy rate for lesions ≤ 4 cm or lesions sized 2–4 cm (*p* = 0.135 and *p* = 0.075, respectively). The systems with the lowest and highest unnecessary biopsy rate for lesions ≤ 4 cm were ACR-TIRADS (62.2%) and K-TIRADS (89.7%), respectively. The unnecessary biopsy rates (≤ 4 cm) were significantly different among the six systems (*p* < 0.001). ACR-TIRADS (62.2%) and C-TIRADS (70.5%) had lower unnecessary biopsy rates than the other systems.

## Discussion

Our study showed that the stratification systems did not help distinguish FTA from FTC. In addition, while the systems showed acceptable performance for correctly determining the indication for biopsy in FTC, the performance with respect to reducing unnecessary biopsy was unsatisfactory.

In this study, on using high or intermediate suspicious stratification as the positive cutoff for FTC, the AUCs of all six systems (0.511 to 0.611) were less than 0.700. A Korean study that focused on the performance of K-TIRADS in classifying follicular neoplasms found the low efficiency of K-TIRADS using the same cutoff (AUC = 0.575, *p* = 0.439) [19]. However, Liu et al reported acceptable performances of 2015 ATA (AUC = 0.744, *p* < 0.001) and ACR-TIRADS (AUC = 0.744, *p* < 0.001) for distinguishing follicular neoplasms [20]. In our study, AUCs of K-TIRADS, EU-TIRADS, ACR-TIRADS, and C-TIRADS were disappointing (AUC = 0.573–0.611, *p* < 0.05). It may be difficult to improve clinical management of nodules based on these systems. The poor performance is attributable to the fact that follicular neoplasms present with substantially overlapping ultrasound features, and FTCs rarely present with features favoring malignancy as described in the current systems, such as nonparallel, markedly hypoechoic, irregular margin, and microcalcifications [3, 22, 23]. A recent meta-analysis identified sonographic features for differentiating between FTA and FTC and found that tumor protrusion, presence of calcifications (irrespective of type), irregular margins, marked hypoechogenicity, and irregular shape were associated with high risk of FTC [24]. Our group found similar high-risk features, such as presence of calcifications (10.7% vs 20.84%, *p* < 0.001) and irregular shape (1.1% vs 14.9%, *p* < 0.001). Therefore, future systems may need to reassess the significance of these features in distinguishing follicular neoplasms.

In addition, our study found that “pattern-based” systems (2015 ATA, AACE/ACE/AME, K-TIRADS, and EU-TIRADS) had more limitations in the classification of follicular neoplasms than previously reported in the literature [19–21], in contrast to the “score-based” systems (ACR-TIRADS and C-TIRADS). Given the limitations of pattern-based systems in the classification of follicular neoplasms, these systems should incorporate findings, such as undetermined composition, irregular shape, iso/hyperechoic with microcalcification and ill-defined margin (Table 4), or switch to “score-based” systems in the future.

In our cohort, the missed biopsy rates for all FTCs ranged from 9.0 to 22.4% and for FTCs ≤ 4 cm ranged from 16.2 to 35.1%, which is concordant with the study by Castellana et al [21] in which 0 to 31% of FTCs were missed. Therefore, we believe that all six systems assessed in this study are effective tools to select FTC for FNA in clinical practice. In fact, previous studies have shown that follicular neoplasms are large [22–27]. The mean maximum diameter of FTC (3.9 ± 2.1 cm) in our study was higher than any threshold proposed for ultrasound-based malignancy risk stratification systems. The highest maximum diameter was up to 12.1 cm, which is the main reason for the high performance in selecting FTC for FNA. Besides, the size of FTC at the time of management is important. FTCs larger than 2 cm have a higher risk of distant metastasis and are associated with worse prognosis [28]. In our study, a satisfactory performance (0 to 20.0%) was also seen for FTCs sized 2 to 4 cm. Apart from unclassifiable nodules, missed FTCs were classified as category 2 and 3 by ACR-TIRADS, and as category 3 by C-TIRADS. There were 4 FTC lesions that were missed by both systems, including a solid isoechoic nodule and three mixed (predominantly solid) isoechoic nodules, which are generally considered indicative of benignity. Therefore, meticulous care should be exercised while managing non-high risk nodules as well.

However, correctly determining the indication for biopsy is only a step towards further diagnostic workup, since the definitive distinction between follicular neoplasms is only based on postoperative histopathology [3]. The risk of malignancy associated with a FNA reading of Bethesda IV (follicular neoplasm or suspicious for a follicular neoplasm) is 10–40% and that with Bethesda III (atypia of undetermined significance or follicular lesion of undetermined significance) is 6–18% [29]. The possibility of FTC in lesions with the above suspicious malignant cytological findings is uncertain. In previous studies, 10.8% follicular neoplasm (FN) and 1.2% follicular lesion of undetermined significance (FLUS) were eventually found to be FTCs [30, 31]. As an extreme example, 1379 thyroid nodules with FNA findings consistent with FN were not diagnosed as FTC after surgery [32]. Other studies have shown that the efficacy of TIRADS depends on the incidence of PTC in the study population [33] and that suspicious ultrasound features may not be useful in predicting malignancy of FLUS [34]. Therefore, due discretion is required due to the limitations of cytology in the diagnosis of FTC.

In previous studies involving papillary carcinoma as the primary malignant tumor, unnecessary biopsy rates with ultrasound-based malignancy risk stratification systems were generally lower than 50% [14–17]. However, our study found higher unnecessary biopsy rates in patients with follicular neoplasm. In our study, FNA was considered indicated for 65.3% to 93.1% of all FTAs and for 62.2% to 89.7% of FTAs sized ≤ 4 cm. We believe that this result is mainly due to the large size of follicular neoplasms [22–27]. Furthermore, our study showed that the unnecessary biopsy rates with use of “pattern-based” systems were higher than those with use of “score-based” systems irrespective of the lesion size because of the different criteria for determining the indications for biopsy. According to “pattern-based” systems, FNA is not indicated only when nodules with special patterns exceed the above size threshold [8–11], such as entirely spongiform and pure cyst nodules (3.3% and 0% of FTAs, respectively). In “score-based” systems [12, 13], FNA is not indicated for nodules that are classified as category 1 and 2 by ACR-TIRADS or as category 2 and 3 by C-TIRADS, irrespective of the size. In our study, a certain proportion of FTAs was categorized in the “no biopsy” group by the ACR-TIRADS and C-TIRADS (23.6% and 26.4% of FTAs, respectively).

Currently, there is an impetus on reducing unnecessary biopsy of thyroid nodules, because of the low rate of malignancy and the generally good prognosis of the most common thyroid malignancy (PTC) [8]. Nevertheless, FTC exhibits a more aggressive biological behavior than PTC. Although the precise diagnosis of follicular neoplasms requires postoperative histopathology, preoperative biopsy and further molecular testing may provide supplementary information aiding the differential diagnosis of follicular neoplasms [35]. Thus, deliberately avoiding biopsy and further testing for follicular neoplasms require careful consideration. Recently, some studies have demonstrated the value of molecular testing for follicular neoplasms. For example, a study using next-generation sequencing reported that the presence of FLT3 and TP53 with no RET mutations was consistent with FTC and the absence of FLT3 and TP53 with the presence of RET mutations was consistent with FTA [36]. Another study investigating DNA methylation haplotype block markers identified 70 DNA methylation markers that were significantly different between the FTC and FTA samples [37].

There are several limitations of our study. First, this was a single-center retrospective study. Only patients with a confirmed postoperative diagnosis of thyroid follicular neoplasm were included. Patients who did not undergo surgery were missed, which may have introduced an element of selection bias. Additionally, 126 nodules (27.7%, 126 of 455) were excluded which may also have resulted in selection bias. Finally, only non-dynamic images were available for recording the ultrasound features which may have affected the accuracy of data.

In conclusion, the currently used malignancy risk stratification systems for thyroid nodules showed poor ability in distinguishing FTA from FTC. The performance of these systems in selecting nodules for biopsy was acceptable, but the performance with respect to reducing unnecessary biopsy was unsatisfactory. Our findings indicate the need to develop a specific stratification system and recommendations for follicular neoplasms.

## Supplementary Information

Below is the link to the electronic supplementary material.

Supplementary file1 (DOCX 31 KB)

## Abbreviations

2015 ATA: 2015 American Thyroid Association Management Guidelines for Adult Patients with Thyroid Nodules and Differentiated Thyroid Cancer
AACE/ACE/AME: American Association of Clinical Endocrinologists, American College of Endocrinology, and Associazione Medici Endocrinology Medical Guidelines for Clinical Practice for the Diagnosis and Management of Thyroid Nodules (2016 Update)
ACR-TIRADS: American College of Radiology Thyroid Imaging Reporting and Data System
AUC: Areas under the receiving operator characteristics curve
AUS: Atypia of undetermined significance
CI: Confidence intervals
C-TIRADS: 2020 Chinese Guidelines for Ultrasound Malignancy Risk Stratification of Thyroid Nodules
EU-TIRADS: European Thyroid Association Guidelines for Ultrasound Malignancy Risk Stratification of Thyroid Nodules in Adults
FLUS: Follicular lesion of undetermined significance
FN: Follicular neoplasm
FNA: Fine-needle aspiration
FTA: Follicular thyroid adenoma
FTC: Follicular thyroid carcinoma
K-TIRADS: Revised Korean Society of Thyroid Radiology Consensus Statement and Recommendations
NPV: Negative predictive value
PPV: Positive predictive value
PTC: Papillary thyroid carcinoma
SD: Standard deviation
SFN: Suspicious for a follicular neoplasm
TIRADS: Thyroid Imaging Reporting and Data System

## Declarations

### Guarantor

The scientific guarantor of this publication is Jian Kuang.

### Statistics and biometry

One of the authors has significant statistical expertise.

### Methodology

- •retrospective
- •diagnostic or prognostic study/observational
- •performed at one institution
