# Supplementary material for: Performance of current ultrasound-based malignancy risk stratification systems for thyroid nodules in patients with follicular neoplasms
Source: Eur Radiol. 2022 Jan 1;32(6):3617–30. doi: 10.1007/s00330-021-08450-3 (PMC9122875; doi:10.1007/s00330-021-08450-3)
Supplement: Supplementary file 1 — Supplementary file1 (DOCX 31 KB) [file 330_2021_8450_MOESM1_ESM.docx]

| **Supplementary 1. Current** **ultrasound-based malignancy risk stratification systems for thyroid nodules with their expected prevalence of malignancy and thyroid biopsy threshold** | | | |
| --- | --- | --- | --- |
| **Ultrasound-based malignancy risk stratification systems** | | **ROM** | **Biopsy threshold** |
| ATA | Benign | 0 | No biopsy |
|  | Very low suspicion | <3% | ≥2.0cm |
|  | Low suspicion | 5-10% | ≥1.5cm |
|  | Intermediate suspicion | 10-20% | ≥1.0cm |
|  | High suspicion | 70-90% | ≥1.0cm |
| AACE/ACE/AME | Low | 1% | >2.0cm |
|  | Intermediate | 5-15% | >2.0cm |
|  | High suspicion | 50-90% | >1.0cm |
| K-TIRADS | Benign^a^ (K-TR2) | <3% | ≥2.0cm |
|  | Low suspicion (K-TR3) | 3-15% | ≥1.5cm |
|  | Intermediate suspicion (K-TR4) | 15-50% | ≥1.0cm |
|  | High suspicion (K-TR5) | >60% | ≥1.0cm |
| EU-TIRADS | Benign (EU-TR2) | 0 | No biopsy |
|  | Low risk (EU-TR3) | 2-4% | >2.0cm |
|  | Intermediate risk (EU-TR4) | 6-17% | >1.5cm |
|  | High risk (EU-TR5) | 26-87% | >1.0cm |
| ACR-TIRADS | Benign (ACR-TR1) | <2% | No biopsy |
|  | Not suspicious (ACR-TR2) | <2% | No biopsy |
|  | Mildly suspicious (ACR-TR3) | 5% | ≥2.5cm |
|  | Moderately suspicious (ACR-TR4) | 5-20% | ≥1.5cm |
|  | Highly Suspicious(ACR-TR5) | >20% | ≥1.0cm |
| C-TIRADS | C-TR2 | 0 | No biopsy |
|  | C-TR3 | <2% | No biopsy |
|  | C-TR4A | 2-10% | >1.5cm |
|  | C-TR4B | 10-50% | >1.0cm |
|  | C-TR4C | 50-90% | >1.0cm |
|  | C-TR5 | >90% | >1.0cm |

1. The Partially cystic nodule with comet-tail artifact is not applicable for FNA according to K-TIRADS.

Abbreviations: ROM: risk of malignancy; ATA: 2015 American Thyroid Association Management Guidelines for Adult Patients with Thyroid Nodules and Differentiated Thyroid Cancer; AACE/ACE/AME: American Association of Clinical Endocrinologists, American College of Endocrinology, and Associazione Medici Endocrinology Medical Guidelines for Clinical Practice for the Diagnosis and Management of Thyroid Nodules (2016 Update); EU-TIRADS: European Thyroid Association Guidelines for Ultrasound Malignancy Risk Stratification of Thyroid Nodules in Adults; K-TIRADS: Revised Korean Society of Thyroid Radiology Consensus Statement and Recommendations; ACR-TIRADS: American College of Radiology Thyroid Imaging Reporting and Data System; C-TIRADS: 2020 Chinese Guidelines for Ultrasound Malignancy Risk Stratification of Thyroid Nodules; FNA: Fine Needle Aspiration.

| **Supplementary 2. Demographic characteristics and ultrasound features of 329 follicular thyroid neoplasms based on pathological classifications** | | | | | | | | |
| --- | --- | --- | --- | --- | --- | --- | --- | --- |
|  |  | FTA | FTC | *P* | Minimally invasive | Encapsulated angioinvasive | Widely invasive | *P* |
| **Demographic characteristics** | |  |  |  |  |  |  |  |
| Male |  | 96(36.6) | 17(25.4) | P=0.085 | 11(22.9) | 4(33.3) | 2(28.6) | P=0.665 |
| Age (years) |  | 43.3±13.9(3-82) | 44.1±15.6(10-79) | P=0.623 | 41.2±13.7(10-69.0) | 53.8±19.3(32-79) | 49.3±15.2(29-75) | P=0.131 |
| **Ultrasound features** | |  |  |  |  |  |  |  |
| Maximum diameters (cm)^a^ |  | 3.8±1.5(0.7-9.2) | 3.9±2.1(0.6-12.1) | P=0.658 | 3.4±1.9(0.6-10.0) | 5.8±2.3(3.5-12.1) | 3.8±1.7(2.4-6.8) | P=0.001^d^ |
| Maximum diameters＞2cm^a^ |  | 231(88.2) | 54(81.8) | P=0.219 | 36(75.0) | 12(100) | 6(100) | P=0.077 |
| Maximum diameters＞4cm^a^ |  | 106(40.5) | 29(43.9) | P=0.675 | 17(35.4) | 10(83.3) | 2(33.3) | P=0.008^e^ |
| Location | Left | 133(50.8) | 34(50.7) | P=1.000 | 24(50.0) | 6(50.0) | 4(57.1) | P=0.437 |
|  | Right | 125(47.7) | 32(47.8) |  | 24(50.0) | 5(41.7) | 3(42.9) |  |
|  | Isthmus | 4(1.5) | 1(1.5) |  | 0(0) | 1(8.3) | 0(0) |  |
| Composition^b^ | Solid | 190(72.8) | 61(92.4) | P=0.001 | 43(89.6) | 12(100) | 6(100) | P=0.739 |
| Echogenicity | Hypoechoic | 40(15.3) | 18(26.9) | P=0.026 | 13(27.1) | 3(25.0) | 2(28.6) | P=1.000 |
| Margin | Smooth | 251(95.8) | 52(77.6) | P<0.001^c^ | 40(83.3) | 8(66.7) | 4(57.1) | P=0.100 |
|  | Ill-defined | 11(4.2) | 14(20.9) |  | 8(16.7) | 3(25.0) | 3(42.9) |  |
|  | Irregular | 0(0) | 1(1.5) |  | 0(0) | 1(8.3) | 0(0) |  |
| Calcifications | Present | 28(10.7) | 19(28.4) | P<0.001 | 14(29.2) | 4(33.3) | 1(14.3) | P=0.755 |
| Microcalcifications | Present | 8(3.1) | 8(11.9) | P=0.003 | 6(12.5) | 2(16.7) | 0(0) | P=0.706 |
| Shape | Irregular | 6(1.1) | 10(14.9) | P<0.001 | 6(12.5) | 2(16.7) | 2(28.6) | P=0.402 |
| Orientation | Nonparallel | 0(0) | 1(1.5) | P=0.204 | 1(2.1) | 0(0) | 0(0) | P=1.000 |
| Peripheral halo | Present | 159(60.7) | 35(52.2) | P=0.210 | 29(60.4) | 4(33.3) | 2(28.6) | P=0.114 |
| Extrathyroidal extension | Present | 0(0) | 1(1.5) | P=0.204 | 0(0) | 1(8.3) | 0(0) | P=0.284 |
| Suspicious cervical lymph node | Present | 0(0) | 3(4.5) | P=0.008 | 0(0) | 2(16.7) | 1(14.3) | P=0.020^f^ |
| The location of the solid component for mixed-content nodules | eccentric | 17(25.4) | 4(80.0) | P=0.023 | 4(80.0) | - | - | - |
| Hyperechoic foci | Comet-tail artifacts | 4(1.5) | 0(0) | P=0.586 | 0(0) | 0(0) | 0(0) | - |
|  | Indeterminate | 2(0.8) | 0(0) | P=1.000 | 0(0) | 0(0) | 0(0) | - |
| Vascularization | absent | 11(4.2) | 0(0) | P=0.090 | 0(0) | 0(0) | 0(0) | P=1.000 |
|  | perinodular | 3(1.1) | 3(4.5) |  | 3(6.3) | 0(0) | 0(0) |  |
|  | intranodular | 5(1.9) | 1(1.5) |  | 1(2.1) | 0(0) | 0(0) |  |
|  | mixed | 243(92.8) | 65(94.0) |  | 44(91.6) | 12(100) | 7(100) |  |

Values are presented as number (%) or mean±SD (range).

Continuous variables were compared using independent two-sample *t* test or rank-sum test (for multiple samples and heterogeneity of variance).

Nominal variables were compared using Chi-squared test or Fisher’s exact test (for small cell values).

a. The maximum diameters could not be determined in a case of widely invasive FTC due to macrocalcifications.

b. Of two cases undetermined the compositions due to calcifications, one was a FTA and the other was a widely invasive FTC.

c. There was a significant difference in the risk of FTC between smooth and ill-defined margin nodules (P<0.05).

d. There was an intergroup difference between the minimally invasive and the encapsulated angioinvasive group (P=0.001).

e. There was an intergroup difference between the minimally invasive and the encapsulated angioinvasive group (P<0.05).

f. Intergroup differences were found between the minimally invasive and the encapsulated angioinvasive group, also between the minimally invasive and the widely invasive group, respectively (P<0.05).

Abbreviations: FTA: Follicular Thyroid Adenoma; FTC: Follicular Thyroid Carcinoma.
